# Deposit Diseases as Differential Diagnosis of Left Ventricular Hypertrophy in Patients with Heart Failure and Preserved Systolic Function

**DOI:** 10.36660/abc.20180370

**Published:** 2019-11

**Authors:** Fábio Fernandes, Murillo Oliveira Antunes, Viviane Tiemi Hotta, Carlos Eduardo Rochitte, Charles Mady

**Affiliations:** 1 Instituto do Coração do Hospital das Clínicas da Faculdade de Medicina da Universidade de São Paulo, São Paulo, SP - Brazil; 2 Fleury Medicina e Saúde, São Paulo, SP - Brazil

**Keywords:** Heart Failure, Hipertrophy, Ventricular, Cardiomyopaty, Restrictive, Amyloidosis, Fabry Disease

Heart failure with preserved systolic function is the main clinical manifestations of patients with ventricular hypertrophy. Conventional treatment is based on the improvement of diastolic dysfunction and congestion. However, no drug has been shown to be effective in the survival of these patients. Thus, it is important to look for the etiology of ventricular hypertrophy with the aim of a treatment directed to the underlying disease.

Among patients with heart failure with preserved systolic function and increased ventricular wall thickness, the clinician should consider as possible differential diagnoses: hypertensive heart disease, deposit disease and hypertrophic cardiomyopathy (Figure 1).^1^

**Figure 1:**
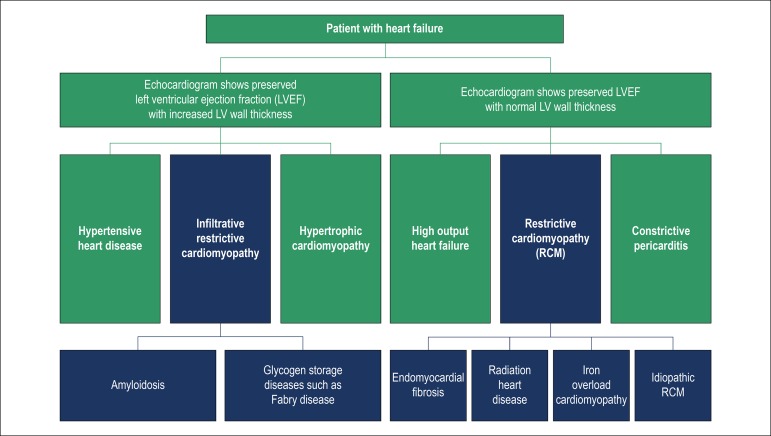
Diagnostic flow chart heart failure patients with and without left ventricular hypertrophy.^1^

Restrictive cardiomyopathies are the least common forms of heart muscle disease. They can be characterized as infiltrative and non-infiltrative, deposit disease or endomyocardial disorders. Possible restrictive cardiomyopathies that may mimic and even be diagnosed as hypertrophic cardiomyopathy are: Amyloidosis, Fabry disease (FD) and Glycogen deposit disease.^1^

We have observed in our group that many patients with deposit disease have been followed for years with a diagnosis of left ventricular hypertrophy (LVH) or hypertrophic cardiomyopathy.

A non-systematic literature review was performed to address the principal papers that suggest deposit diseases such as ventricular hypertrophy etiology and the “red flags” for a possible diagnosis. The database consulted was PubMed (www.ncbi.nlm.nih.gov/pubmed). Original articles and review performed in humans, written in Portuguese and English, were selected and the keywords MeSH hypertrophic cardiomyopathy, amyloidosis, Fabry's disease and glycogen deposit disease were used as keywords. In the present literature review, we will first address amyloidosis and later FD and finally glycogen deposit disease.

Amyloidosis is a disease caused by the deposition of amyloid fibrils in various organs,^2^ including the heart, where amyloid proteins infiltrate the ventricular wall, with consequent thickening causing systolic and diastolic dysfunction, heart failure (HF) and conduction disorders, with high mortality.^2-4^

More than 30 proteins can form amyloidosis, and among these, five can affect the heart. However, the most common types of amyloidosis that infiltrate the heart are: 1) light chain immunoglobulin, also called primary amyloidosis or AL amyloidosis;^5^ 2) Transthyretin called amyloidosis-ATTR, which may be of genetic origin also called familial form ATTRm and form ATTRwt, also called wild type.

Detection and differentiation between these two forms are fundamental because they have different treatment and clinical evolution. Some patients with ATTRm and ATTRwt may have increased serum immunoglobulins, which may confuse the clinician regarding the correct type of amyloidosis.

The importance of the proper diagnosis of amyloidosis is based on that the treatment is different from other forms of heart failure and hypertrophic cardiomyopathy. In patients with amyloidosis, digitalis, calcium blockers and high doses of beta-blockers should be avoided, in addition, there are currently new therapeutic possibilities related to the underlying disease.^6^

The goal of amyloidosis treatment is to stop protein production by reducing the burden of circulating amyloid proteins.^6-8^

In AL amyloidosis there is a direct toxic effect of circulating immunoglobulin that may contribute to myocardial dysfunction and lead to early diastolic dysfunction. This may explain the discrepant findings between the severity of symptoms and changes in diastolic function in patients with little or no thickening evaluated by echocardiography. Patients have seen five clinicians with an average of two years before the correct diagnosis of AL amyloidosis and when this is often made the prognosis is reserved. Therefore, it is important for clinicians to consider AL amyloidosis as a differential diagnosis of hypertrophy or heart failure with preserved systolic function.^1,8^

More than 150 mutations are described in genetic form related to the ATTR protein gene. The most common allele in the US is Val122Ile, found in 4% of African American individuals. In our country, the change of valine by methionine to position 30 is the most common mutation (Val30Met). In countries like Japan, there is a bimodal pattern of presentation in this mutation (Val30Met): an early form that occurs around 20 to 40 years, characterized by loss of thermal and sensory sensitivity, family history, high penetrance, autonomic dysfunction and conduction disorders. In the late form, which begins around 50 years of age, patients have sensory-motor symptoms in the lower distal extremities, low penetrance, mild dysautonomia and greater myocardial impairment. The presence of neurological or systemic symptoms should suggest to clinicians as red flags to the possibility of ATTRm amyloidosis.^3,4,7^

The wild form, formerly called senile, as it can also affect individuals under the age of 50, predominantly affects the heart. It usually affects males with aged 77 years and usually there are neurological symptoms, such as carpal tunnel and spinal canal stenosis, five years before cardiac symptoms. Wild amyloidosis is more prevalent than we previously thought and can be detected at necropsy in up to 20% of individuals with preserved systolic heart failure (HFPEF) and in 13% of HFPEF patients hospitalized with ventricular wall thickening over 12 mm.^9,10^

Patients with TTR amyloidosis have ventricular wall thickening, diastolic dysfunction, and conduction system disorders.^6,11^ Although, these patients have better survival when compared to patients with AL amyloidosis. ATTR-amyloidosis progresses when not treated properly with heart failure (HF), reduced functional capacity, and severe arrhythmias.^12^

We have observed in our group that patients with ATTRwt have been treated as hypertensive heart disease or HFPEF. The presence of left ventricular hypertrophy disproportionate to the degree of hypertension, previous motor sensory symptoms and the presence of dysautonomia may be the red flags for a possible diagnosis of deposit disease.

Degenerative aortic calcification occurs in elderly individuals over 75 years and may evolve with signs and symptoms of HF. However, in a necropsy study, the presence of cardiac amyloidosis and aortic stenosis has been observed in this population.^13^

Amyloidosis causes diastolic dysfunction and ventricular thickening that shares some features with aortic stenosis. The concomitance of ATTRwt and aortic stenosis may lead to significant ventricular hypertrophy and functional impairment that may be confused with low flow, low gradient aortic stenosis. Amyloid deposition may lead to the use of pacemaker after percutaneous procedures (TAVI) and high prevalence of late enhancement evaluated by cardiac magnetic resonance imaging.^14^

Treibel et al.^14^ studying patients with severe aortic stenosis found a prevalence of amyloidosis in 6% of cases, all with ATTRwt. In these patients, there was a higher mortality, about 50%, and the authors suggest that nuclear scintigraphy could be a complementary method in the diagnosis of amyloidosis and could influence the need for interventional treatment and the use of specific therapies for amyloidosis. We have observed in our group that some patients despite interventional aortic valve treatment remain symptomatic and that the real aetiology for the symptoms was ATTRwt.

Deposits of amyloid may also be found in a histopathological study of patients with hypertrophic cardiomyopathy undergoing myectomy surgery.^15,16^

TTR mutation may be common in individuals > 55 years diagnosed as HCM, particularly African Americans. Danny et al evaluated 298 patients from 9 French centers the presence of amyloidosis in patients with ventricular hypertrophy over 15 mm. Mutation genotype of ATTRm was found in 17 patients. The prevalence of ATTRm was 5% and 8.3% in patients > 55 years, respectively. The mutations found were: V142I (8), V50M (2) and I127V (2). Patients with ATTRm mutation were older, had a higher frequency of neuropathy (53%), carpal tunnel syndrome (46%), low ECG voltage (36%), symmetrical hypertrophy (92%), abnormal left ventricular function, increased filling pressures and late gadolinium enhancement when compared to patients without the mutation. Thus, amyloidosis should be considered as a differential diagnosis in patients with hypertrophic heart disease.^17^ In our group, four patients initially diagnosed with HCM had the diagnose of ATTRm form with V142I mutation.

Amyloidosis should be suspected in patients with bilateral carpal tunnel syndrome, unexplained neuropathic pain, orthostatic hypotension, and hypertrophic cardiomyopathy diagnosed after the 6th decade of life.^6^

Complementary methods (ECHO and CMR) contribute to the recognition of cardiac amyloid infiltration, degree of ventricular hypertrophy and systolic and diastolic dysfunction.^18,19^However, these morphological and functional changes represent an advanced picture of disease and correlate it with the amount of amyloid in the whole body.^20^ This stage of the disease is also related to worsening clinical signs and symptoms.^21^

Typical echocardiographic findings are: Left ventricular thickness with right ventricular (RV) involvement, decreased biventricular longitudinal axis function with normal or near normal ejection fraction (EF) and increased valve thickness.^22^and blood flow assessment by Doppler allows also the identification of cardiac hemodynamics present in the restrictive form.^23,24^

Patients with ATTRwt had a higher left ventricular thickness and lower ejection fraction and the longitudinal strain is lower in ATTRwt and AL forms when compared to ATTRm.^2^(Figure 3, 4 and 5)

**Figure 2:**
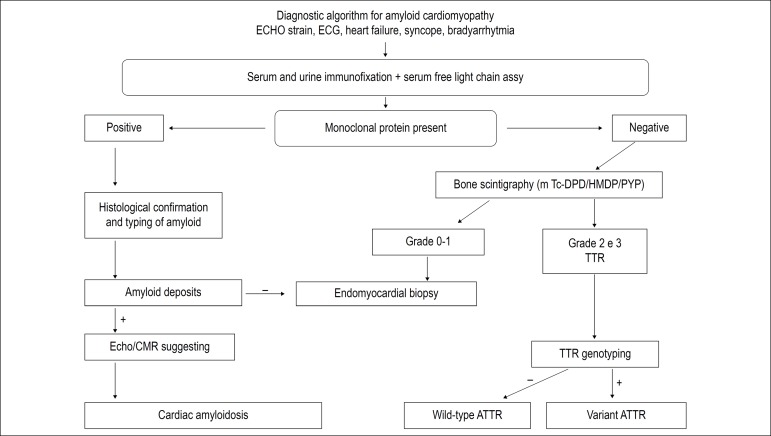
Evaluation of patients with suspected cardiac amyloidosis.^1^

**Figure 3:**
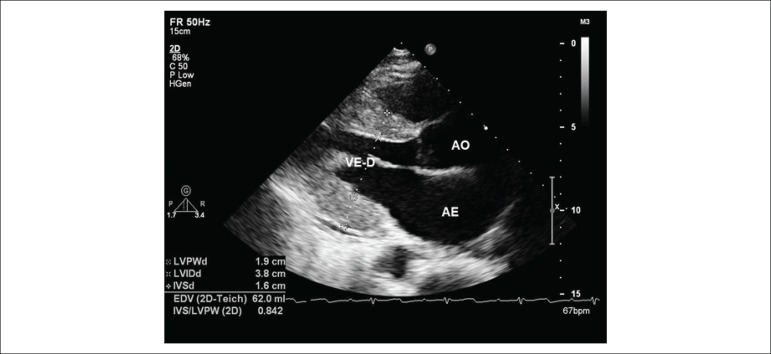
Image obtained from transthoracic echocardiography. Longitudinal parasternal section showing the increased myocardial thickness of the anterior and inferior lateral septal walls of a patient with AL form amyloidosis. Bright aspect of the myocardial walls suggestive of infiltrative disease is observed.

**Figure 4:**
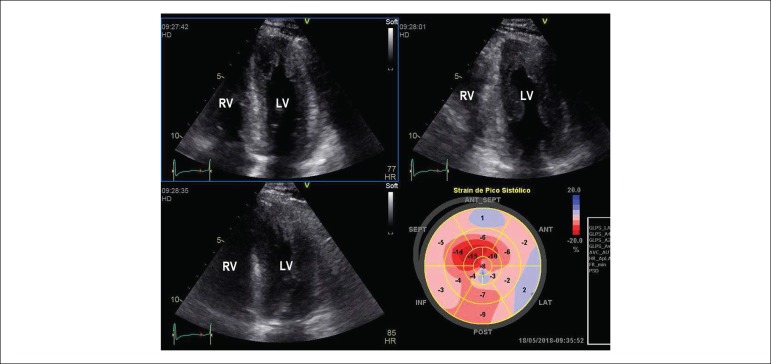
Image obtained from transthoracic echocardiography. 4-chamber apical section showing a diffuse increase in the thickness of the left ventricular myocardial walls. Below, on the right, there is a parametric image of the longitudinal myocardial deformation evaluation by the speckle tracking technique.

**Figure 5:**
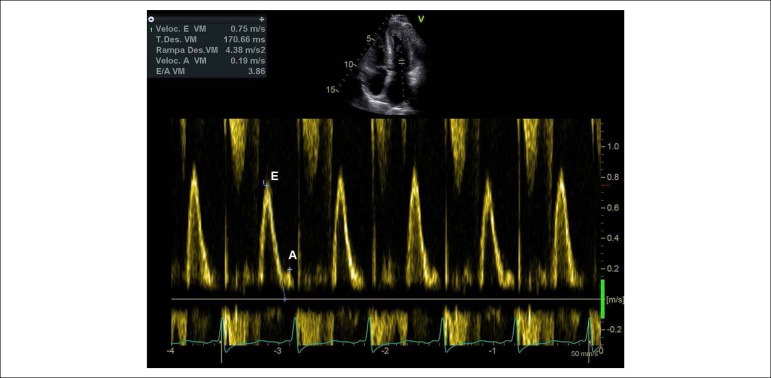
Image obtained from transthoracic echocardiography. Mitral Doppler spectral curve of a patient with amyloidosis showing a restrictive diastolic pattern (E/A ratio > 2)

More advanced ECO techniques such as strain and strain rate derived from speckle tracking may assist in the assessment of cardiac torsion movements and facilitate the differentiation between cardiac amyloidosis and hypertrophic cardiomyopathy. In patients with amyloidosis there is a regional variation from the base to the apex to the longitudinal strain, where the apical portion is preserved, and this accurate and reproductive pattern in the differentiation between cardiac amyloidosis and other forms of left ventricular hypertrophy.^18^ LV strain analysis at rest is an independent predictor of mortality from both cardiac and other causes.^25^

Cardiac magnetic resonance imaging (CMR) provides information on cardiac function and morphology in patients with amyloidosis.^19^ However, CMR is better for assessing and quantifying abnormalities in ventricular diastolic function. The images from CMR allow 3-dimensional evaluation of cardiac volumes, cardiac wall thickening and mass. In addition, new techniques used by CMRI such as gadolinium late enhancement are fundamental in identifying cardiac amyloid infiltrates and enable the differentiation between a patient with amyloidosis and patients with ventricular hypertrophy, such as patients with hypertension and hypertrophic cardiomyopathy. However, the clinician should consider the high degree of clinical suspicion combined with complementary imaging methods, as often the anatomical features of hypertrophy can simulate both diseases.^26^

The asymmetric pattern of myocardial hypertrophy in patients with ATTR amyloidosis differs from AL patients, usually symmetrical. In a study of 263 patients with TTR amyloidosis confirmed by grade 2 myocardial scintigraphy and compared with 50 patients with AL form, it was observed: in the TTR form there was asymmetric hypertrophy in 79% of cases, symmetrical in 18% and 3% without LVH. The late enhancement pattern was 29% subendocardial and 71% transmural.^26^

We can identify three stages of disease by resonance with different prognosis: phase 1 - no evidence of late enhancement, but with increased extracellular volume and T1 map with 92% survival at 24 months; phase 2 - increased extracellular volume and T1 map and appearance of late subendocardial enhancement with 81% survival; and phase 3 - increased extracellular volume and T1 map and progression to late transmural enhancement with 61% survival. In multivariate analysis, the presence of transmural late enhancement increased mortality 4-fold and extravascular space correlated with amyloid loading and was an independent prognostic marker of survival.^19^

Technetium bone scintigraphy may be useful for differentiating the AL form from TTR. TTR amyloidosis often has a higher number of microcalcifications, which would justify its higher uptake of these radiopharmaceuticals. Using a visual score (0: absence of uptake, 1: uptake less than bone, 2: uptake equal to bone and 3: uptake upwards - more intense than bone uptake) to assess cardiac technetium uptake, it is possible to differentiate the shape TTR of other types of cardiac amyloidosis in conjunction with absence of light chain immunoglobulins in blood and urine. Thus, the diagnosis of TTR amyloidosis can be performed without the need for biopsy.^6^

Figure 2 flowchart for evaluation of patients with suspected cardiac amyloidosis.

Amyloidosis is a heart disease with heterogeneous phenotype and in many cases there are symptoms preceding in years the onset of cardiac manifestations. Thus, clinicians should have a high degree of suspicion to be able to direct the exams and do a correct diagnosis

## Fabry Disease

Fabry's disease FD is a genetic, lysosomal storage disorder caused by total or partial alpha-galactosidase (α-Gal A) deficiency of the enzyme that degrades glycolipid globotriaosylceramide (Gb3) in lysosomes.^27^ Therefore Gb3 deposits accumulate in the endothelial cells and heart, resulting in organic dysfunction.^28^ The gene that causes FD is located in the long arm of the X chromosome (*locus* Xq22) and currently hundreds of pathogenic mutations have been described.^29^ Thus, male homozygous patients develop the classic disease, while female heterozygous patients present variable clinical manifestations from conditions without apparent clinical disease to the complete expression of the disease.^29-31^

In clinical practice, early diagnosis is important because actually there are specific treatment with enzyme replacement therapy (ERT) that may change the natural history, reducing and/or stabilizing the progression of the disease.^32,33^

The FD cardiac phenotype is left ventricular hypertrophy (LVH) and FD should be considered as a differential diagnosis of hypertrophic cardiomyopathy Some studies have shown that up to 5% of these patients would have the diagnosis of FD.^34-36^ We have observed in our outpatient clinic patients with FD and onset of hypertrophy in adulthood, a progressive character, with electrocardiographic alterations and echocardiographic findings similar to those of HCM, including LV outflow tract obstruction. Just like amyloidosis, there are also systemic manifestations that may infer the diagnosis of FD.^34-36^

Gb3 deposits are present in all cellular components of the myocardium, such as cardiomyocytes, conduction system, valvular fibroblasts, endothelial cells and vascular smooth muscle cells, but their totality represents only 1% to 2% of all cardiac mass, suggesting activation of other signaling pathways leading to hypertrophy, apoptosis, necrosis and fibrosis.^37^

Concentric ventricular hypertrophy is the most typically found in FD, but approximately 5% of cases present as asymmetric septal hypertrophy with dynamic LV outflow tract obstruction. Although LVH has been detected in some children, cardiovascular signs and symptoms are usually present in the third or fourth decade of life in men and one decade later in women.^38,39^ The presence of LVH leads to a reduction in life expectancy by approximately 20 years in men and 15 years in women when untreated compared to the general population.^40,41^

The magnitude of hypertrophy increases with age and is inversely related to renal function and α-Gal A activity. Right ventricular involvement is common with no functional or clinical consequences.^42^ Cardiac manifestations may occur as the only manifestation of the disease called “cardiac variant”.^43^

The diagnosis of myocardial hypertrophy is performed by echocardiography with the presence of bright endocardium or binary appearance of the border of the endocardium. This fact represents the compartmentalization of Gb3 and was proposed as a marker of Fabry's disease.^44^ However, subsequent studies demonstrated limited sensitivity (15%-35%) and specificity (73%-80%).^45,46^ Diastolic dysfunction occurs early more frequently than systolic dysfunction, and before the development of hypertrophy.^47,48^

Delayed gadolinium enhancement is common in patients with FD.^49-51^ The enhancement presenting with a non-ischemic pattern, located in the mesocardium and not affecting subendocardium, in basal and middle segments of the anterolateral and inferolateral walls.^52^ Among men, myocardial fibrosis occurs only in those with ventricular hypertrophy, differently myocardial fibrosis emerge without LVH in women.^38,53^

Other findings are commonly identified in FD patients: mild thickening and mitral and/or aortic valves regurgitation but usually without the need for valve repair.^37,38,54^ Coronary artery disease manifested as angina often occurs in men and women.^55,56^ Atrial arrhythmias, including atrial fibrillation, are common and appear to be age-related. Non-sustained ventricular tachycardia usually associated to LV wall thickness. Conduction abnormalities may be caused by glycolipid deposition in the atrioventricular (AV) node, His bundle, and branches.^57,58^ The short PR interval, particularly in younger patients,^59,60^ and EKG changes compatible with LVH (QRS complex voltages and repolarization change, opposite to other depository diseases with low QRS complex voltages on electrocardiogram. Sinus node dysfunction and atrioventricular blocks result in bradyarrhythmia requiring pacemaker implantation in older patients.^58,60,61^

The definitive diagnosis of FD in male patients is generally confirmed by measuring alpha-Gal A activity of leukocytes.^62^ However, this assay will identify less than 50% female heterozygotes. In female with suspected Fabry disease (and men with marginal levels of alpha-Gal A activity), genetic testing is recommended.^63,64^

The specific treatment for FD is through ERT which, if started as early as possible, as soon as cardiac manifestations are detected and although there is no evidence yet establishing an effect on cardiovascular outcomes, may prevent the disease from developing in young people, and at least slow the progression of multiple organ dysfunction in older patients.^65-69^(Table 1)

**Table 1:** When Fabry's disease suspect

**1. Unexplained left ventricular hypertrophy (LVH)**
• Male gender
• Atypical: diffuse concentric, med-ventricular or free wall
**2. Electrocardiogram**
• PR shortening (< 120 ms)
**3. Clinical manifestations**
• Angiokeratoma
• Orthostatic hypotension, chronotropic incompetence, syncope and/or recurrent dizziness
• Anidrosis or hyperhidrosis
**4. Others**
• Renal insufficiency
• Stroke
• Verticilata cornea

## Glycogen depot disease

Glycogen deposit diseases are inherited metabolic diseases of glycogen metabolism that can affect its synthesis or degradation in muscle, liver and heart tissues.^70^

Danon's disease has an autosomal dominant X-linked character due to LAMP2 enzyme deficiency and the triad of heart failure with hypertrophic cardiomyopathy, skeletal myopathy and mental deficit in male patients and only cardiomyopathy in women.^71^ The phenotype of cardiomyopathy is usually hypertrophic but dilated has also been described. Myopathy is usually mild with proximal weakness of the limb and cervical muscles, and nerve conduction studies show sensory and motor polyneuropathy. In male patients, the mental deficit may be observed in half of the cases and 10% in females with mild symptoms.

Laboratory tests show a rise in serum creatine kinase (CPK) levels from 5 to 10 x normal limits. Electrocardiogram is abnormal in all patients, showing Wolff-Parkinson-White syndrome (WPW), the high voltage on precordial leads, giant negative T waves, atrioventricular block, atrial flutter, atrial fibrillation, bradycardia, abnormal Q waves, and complete left bundle branch block. Echocardiograms show that most patients present a phenotype of concentric hypertrophic cardiomyopathy with impaired left ventricular function.^71^

PRKAG2 syndrome is a rare autosomal dominant inherited disease characterized by cardiac hypertrophy, ventricular pre-excitation, and conduction system abnormalities and increased risk of sudden death.^72^ It is characterized by increased glycogen storage and glucose uptake as opposite to what occurs due to a defect in glycogen degradation. The clinical presentation is ventricular hypertrophy and tachyarrhythmias that can lead to sudden death, conduction tissue disease, severe myocardial hypertrophy, skeletal myopathy and arrhythmias, often related to Wolff-Parkinson-White syndrome. Occasionally, LV systolic dysfunction and high-grade AV block may require pacemaker implantation. The electrocardiographic appearance is a short PR interval in 70% of cases, right bundle branch block, atrioventricular or sinoatrial blocks.

Cardiac hypertrophy can mainly affect the left ventricle, with progressive character accompanied by systolic and diastolic dysfunction with mean ventricular hypertrophy of 24 mm. High voltage in QRS complexes with ventricular repolarization abnormalities is observed even in the absence of left ventricular hypertrophy on echocardiography.

## Conclusions

There are currently over 6,000 rare diseases in the world. Among those that affect the heart, many may be underdiagnosed, or even mistakenly mistaken for heart diseases most commonly seen in clinical practice, such as hypertensive heart disease and hypertrophic cardiomyopathy. The clinician should always ask for if the diagnosis is correct and should review his concepts. The clinician should try to complete all the puzzle pieces. For these purpose, they can visualize or even suggest the correct diagnosis and towards to a specific treatment. We emphasize the saying of Mark Krane: “*A doctor is not required to know everything. It's impossible. But you need to know where to go when you don't have the answer*”.
